# Use of ^68^Ga-PSMA-11 and ^18^F-FDG PET-CT Dual-Tracer to Differentiate Between Lymph Node Metastases and Ganglia

**DOI:** 10.3389/fonc.2021.646110

**Published:** 2021-03-10

**Authors:** Yiping Shi, Lian Xu, Yinjie Zhu, Yining Wang, Ruohua Chen, Jianjun Liu

**Affiliations:** ^1^ Department of Nuclear Medicine, Ren Ji Hospital, School of Medicine, Shanghai Jiao Tong University, Shanghai, China; ^2^ Department of Urology, Ren Ji Hospital, School of Medicine, Shanghai Jiao Tong University, Shanghai, China

**Keywords:** ^68^Ga-PSMA-11, ^18^F-FDG, ganglia, lymph node metastases, prostate cancer

## Abstract

**Purpose:**

Differentiating lymph node metastases (LNM) from peripheral ganglia by physiological prostate-specific membrane antigen (PSMA) uptake is challenging. Two tracers (^68^Ga-PSMA-11 and ^18^F-fluorodeoxyglucose [FDG]) metabolic uptake patterns were evaluated by positron emission tomography-computed tomography (PET-CT), searching for differences that could tell ganglia from LNM.

**Methods:**

Dual ^68^Ga-PSMA-11 and ^18^F-FDG PET-CT data of 138 prostate cancer patients acquired from June 2018 to December 2019 were retrospectively evaluated. Ganglia and LNM with PSMA-11 uptake above local background were analyzed by the location and PSMA-11-PET and FDG-PET maximum standardized uptake value (SUVmax).

**Results:**

PSMA-11-positive ganglia (n = 381) and LNM (n = 83) were identified in 138 and 58 patients, respectively. The LNM SUVmax of PSMA-11-PET (16.4 ± 14.8 vs 2.3 ± 0.7, *P* < 0.001) and FDG-PET (3.3 ± 3.2 vs 1.5 ± 0.5, *P* < 0.001) were higher than in ganglia. The probabilities of being an LNM in the low-potential (PSMA-11-PET SUVmax of <4.1 and FDG-PET SUVmax of <2.05), moderate-potential (PSMA-11-PET SUVmax of >4.1 and FDG-PET SUVmax of <2.05, or PSMA-11-PET SUVmax of <4.1 and FDG-PET SUVmax of >2.05), and high-potential (PSMA-11-PET SUVmax of >4.1 and FDG-PET SUVmax of >2.05) groups were 0.9% (3/334), 44.6% (37/83), and 91.5% (43/47), respectively (*P* < 0.001). The cervical and coeliac ganglia had higher PSMA-11 and FDG uptake than the sacral ganglia (P < 0.001 for all). LNM PSMA-11 and FDG uptake was similar in these three locations.

**Conclusion:**

The FDG-PET and PSMA-11-PET SUVmax, especially when combined, could well differentiate LNM from ganglia. The tracers uptake differed between cervical/coeliac and sacral ganglia, so the lesion location should be considered during image assessment.

## Introduction

Prostate cancer is a common malignant tumor in males (1). Despite initial treatment by radical prostatectomy, biochemical recurrence (BCR) remains a major problem (2). The ability to determine the location and degree of recurrence is of great significance for treatment planning. However, conventional imaging techniques, including magnetic resonance imaging (MRI) and computed tomography (CT) (3), have limited sensitivity. Since 2012, the application of ^68^Ga-prostate-specific membrane antigen (PSMA) positron emission tomography (PET)-CT has significantly improved detection rates in BCR patients (4–7). Various studies showed that 68Ga-PSMA PET-CT detection efficiency is higher than conventional imaging approaches and choline PET (4, 8).

However, PSMA is expressed on prostate cancer cells and many other tissues, both physiologically (9) and pathologically (10). For instance, PSMA is expressed in the salivary glands, submandibular glands, kidneys, spleen, liver, and more. PSMA is also expressed in neovascularization of many solid tumors (11–13). Besides, many studied reported that peripheral nerve ganglia uptake PSMA (14). It has been reported that astrocytes express PSMA physiologically as PSMA is related to their homolog glutamic acid carboxypeptidase III (15, 16). Such a widespread nonspecific PSMA-11 uptake might lead to potential pitfalls in interpreting images.

Therefore, differentiating lymph node metastases from physiological PSMA uptake in peripheral ganglia is a challenge for nuclear medicine physicians. To solve this problem, some strategies have been proposed. For example, performing a careful anatomic correlation by comparing and examining the morphology of the lesions. Banding was correlated with ganglia, while lymph nodes resemble teardrops or nodules (14). Previous studies have shown that ganglia show mild to moderate PSMA-11 uptake and cervical/coeliac ganglia had higher PSMA-11 uptake than sacral ganglia (14). Recently, Alberts et al. found that delayed ^68^Ga-PSMA PET-CT could be used to differentiate ganglia from lymph node metastases, but the overall diagnostic efficiency was not high, with a sensitivity of 73% and specificity of 65% (17). With such diagnostic efficiency, these methods offer no effective mean to tell lymph node metastases from peripheral ganglia. Therefore, new imaging approaches are needed.

^18^F-fluorodeoxyglucose (FDG)-PET has been extensively used to differentiate benign from malignant lesions. Studies have also indicated that ^18^F-FDG has a gain value in partial prostate cancers with a high Gleason grade (18, 19), especially for prostate cancers with negative ^68^Ga-PSMA PET-CT findings (20, 21). However, studies describing the ^18^F-FDG uptake pattern for ganglia and whether ^18^F-FDG PET-CT could be used to differentiate between them and lymph node metastases are lacking. In addition, whether there were ^18^F-FDG uptake differences of ganglia in different anatomical location were also unknown. Therefore, in this study, we performed dual-tracer (^68^Ga-PSMA-11 and ^18^F-FDG) PET-CT to evaluate the metabolic patterns of these tracers according to different anatomical location in lymph node metastases and ganglia. We assumed that the heterogeneous metabolic patterns of ^68^Ga-PSMA-11 and ^18^F-FDG could be used to differentiate between lymph node metastases and ganglia, and there were also differences in ^68^Ga-PSMA-11 and ^18^F-FDG uptake between cervical/coeliac and sacral ganglia which should be considered for better identification.

## Methods

### Participants

The ethics committee of Renji Hospital approved this retrospective study, which used data obtained for clinical purposes. The need for informed consent was waived. The study was performed in accordance with the ethical standards as laid down in the 1964 Declaration of Helsinki and its later amendments. A total of 138 consecutive patients with prostate cancer who underwent both ^68^Ga-PSMA-11 and ^18^F-FDG PET-CT between June 2018 and December 2020 were enrolled. The PSMA ligand was ^68^Ga-PSMA-11. The inclusion criteria were as follows: (a) Prostate cancer patients who underwent ^68^Ga-PSMA-11 PET-CT and ^18^F-FDG PET-CT with less than two weeks in between; (b) patients characteristics, including age, Gleason grade score, prostate-specific antigen (PSA) level, and treatment history were available; (c) prostate cancer treatment was not done during the interval between the two scans. The detailed patients’ characteristics are listed in **Table 1**.

**Table 1:** Patients characteristics (n=138).

Characteristics	No. of Patients
**Age (y)**	
Mean ± SD	69.2 ± 7.4
Range	55-90
**Gleason score**	
6	4
7	69
8	31
9	31
10	3
**Patient type**	
Staging before treatment	65
Biochemical recurrence	73
**PSA level**	
Staging before treatment (IQR)	56.4 (18.5-99.7)
Biochemical recurrence (IQR)	1.1 (0.5-4.1)
**PSMA-11-positive ganglia**	
No. of patients	138
No. of lesions	381
**PSMA-11-positive lymph node metastases**	
No. of patients	58
No. of lesions	83

### Image Evaluation

Two nuclear medicine physicians with ten (LX, reader 1) and eight (RC, reader 2) years of experience in PET-CT interpretation evaluated together the image data and resolved any disagreements by discussion till they reached consensus. Regions of interest (ROI) were placed over the selected ganglia or lymph node metastases. The maximum standardized uptake value (SUVmax) was calculated as follows: maximum pixel value in the decay-corrected ROI activity (MBq/kg)/[the injected ^18^F-FDG or ^68^Ga-PSMA-11 radioactivity (MBq)/body weight (kg)].

Ganglia and adjacent lymph node metastases were grouped according to their anatomic location: cervical, coeliac, or sacral. The main criterion for ganglia was focal ^68^Ga-PSMA-11 uptake that projected onto a structure of typical type and location for sympathetic ganglia, as described previously (14). Lesions that were considered to be suggestive for ganglia or lymph node metastases and exhibited increased ^68^Ga-PSMA-11 tracer uptake relative to local background were counted. To avoid introducing possible bias, the selection criteria for ganglia were as follows: 1) Only the ganglion with the highest PSMA-11 uptake in each anatomical location (cervical, coeliac, or sacral) was selected if more than one PSMA-11-positive ganglion existed. 2) If the anatomical location had no PSMA-11-positive ganglia, it was defined as PSMA-11-negative. The same selection criteria were used to define and select lymph node metastases with increased ^68^Ga-PSMA-11 uptake relative to local background.

### Statistical Analysis

Results are either demonstrated as mean ± SD or as frequencies (%). For comparison of continuous variables, the 2-tailed unpaired Student t test was used. The x2 test was applied to compare nominal variables. All statistical analyses were performed using SPSS 21.0 (IBM Corp., USA), with a two-sided *P*<0.05 considered statistically significant.

## Results

### Ganglia Uptake Patterns

**Figure 1:**
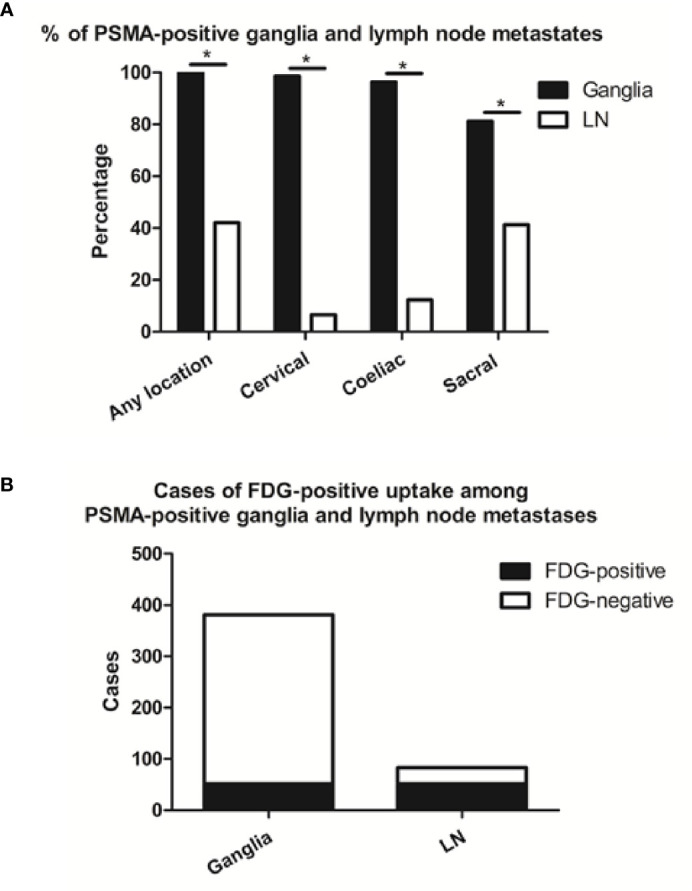
Frequencies of PSMA-11-positive and cases of FDG-positive and -negative ganglia and lymph node (LN) metastases. **(A)** Frequencies of PSMA-11-positive ganglia and lymph node metastases occurring at any location, or limited to the coeliac, cervical, or sacral area on a per-patient-basis. **(B)** Cases of FDG-positive uptake among PSMA-11-positive ganglia and lymph node metastases. Asterisk (*) indicates a significant difference within the same location at *P* < 0.001.

We identified 381 PSMA-11-positive ganglia (i.e., cervical, coeliac, or sacral) in all 138 patients in our cohort (100%), and 83 PSMA-11-positive lymph node metastases in 58 patients (42%; **Table 1**). Grouped by anatomical location, PSMA-11-positive uptake was observed in cervical, coeliac, and sacral ganglia at frequencies of 98.6% (136/138 patients), 96.4% (133/138 patients), and 81.2% (112/138 patients), respectively (**Figure 1A**). Cervical and coeliac ganglia had a higher rate of PSMA-11-positive uptake than sacral ganglia (P < 0.001 for both).

**Figure 2:**
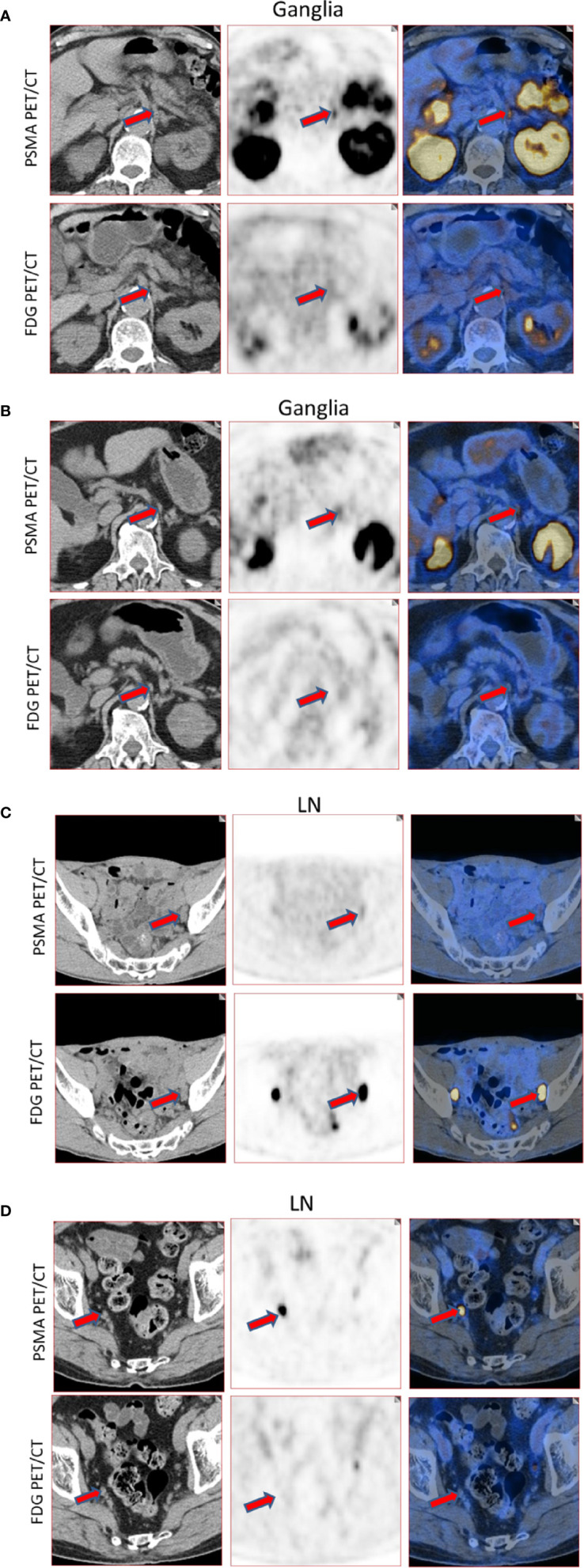
Representative images of PSMA-11-positive ganglia and lymph node metastases. **(A)** FDG-positive celiac ganglia (red arrow, SUVmax of PSMA-11-PET 6.6, SUVmax of FDG-PET 2.9). **(B)** FDG-negative celiac ganglia (red arrow, SUVmax of PSMA-11-PET 3.6, SUVmax of FDG-PET 0.7). **(C)** FDG-positive pelvic lymph node metastasis (red arrow, SUVmax of PSMA-11-PET 3.2, SUVmax of FDG-PET 28.0). Lymph node metastasis was confirmed by postoperative pathology. **(D)** FDG-negative pelvic lymph node metastasis (red arrow, SUVmax of PSMA-11-PET 14.4 and SUVmax of FDG-PET 0.6). Lymph node metastasis was confirmed by postoperative pathology.

**Figure 3:**
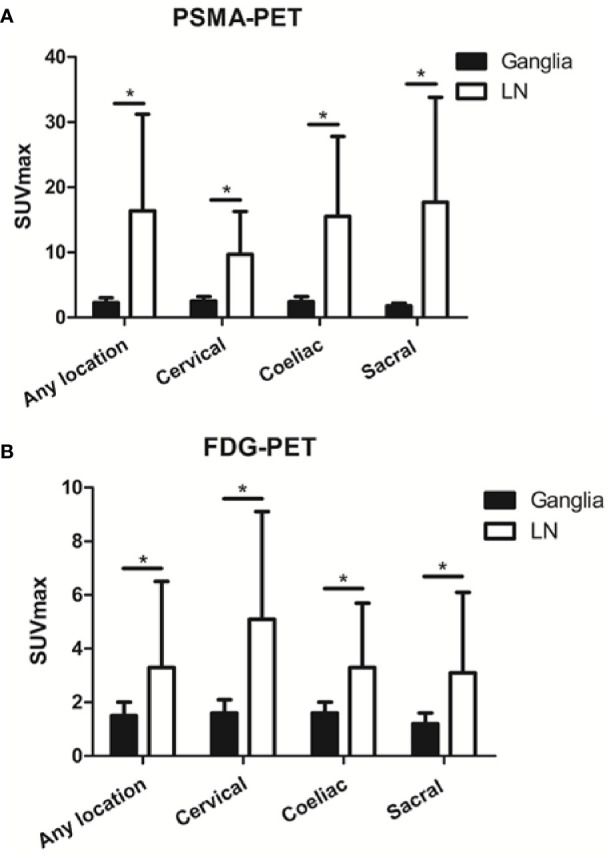
Prostate-specific membrane antigen (PSMA-11) and fluorodeoxyglucose (FDG) uptake in ganglia and adjacent lymph node metastases. **(A)** PSMA-11 uptake in ganglia and adjacent lymph node metastases. **(B)** FDG uptake in ganglia and adjacent lymph node metastases. Asterisk (*) indicates a significant difference within the same location at *P* < 0.001.

Qualitatively, among the 381 PSMA-11-positive ganglia, 13.6% (52/381) were identified as FDG-positive and 86.4% (329/381) as FDG-negative (**Figure 1B**). Quantitatively, the PSMA-11-PET SUVmax ranged from 1.3 to 6.6. The cervical and coeliac ganglia were similar in PSMA-11 uptake (2.5 ± 0.7 vs 2.4 ± 0.8, P = 0.665). However, PSMA-11 uptake in both cervical (2.5 ± 0.7 vs 1.8 ± 0.4, P < 0.001) and coeliac (2.4 ± 0.8 vs 1.8 ± 0.4, P < 0.001) ganglia was significantly higher than in the sacral ganglia (**Figure 3A**). The FDG-PET SUVmax ranged from 0.3 to 3.5. The cervical and coeliac ganglia were similar in FDG uptake (1.6 ± 0.5 vs 1.6 ± 0.4, P = 0.995), but both were significantly higher than in the sacral ganglia (1.6 ± 0.5 vs 1.2 ± 0.4, and 1.6 ± 0.4 vs 1.2 ± 0.4, respectively, P < 0.001 for both; **Figure 3B**). The detailed SUVmax for ganglia and lymph node metastases are listed in **Table 2**. Representative images of ganglia are shown in **Figure 2**.

**Table 2:** SUVmax of PSMA-11-PET and FDG-PET in ganglia and lymph node metastases.

Parameter		Ganglia				LN			
		Any Location	Cervical	Coeliac	Sacral	Any Location	Cervical	Coeliac	Sacral
**PSMA-11**	Mean	2.3	2.5	2.4	1.8	16.4	9.7	15.5	17.7
	SD	0.7	0.7	0.8	0.4	14.8	6.6	12.3	16.1
	Median	2.1	2.4	2.3	1.7	10.4	8.7	8.8	11.7
**FDG**	Mean	1.5	1.6	1.6	1.2	3.3	5.1	3.3	3.1
	SD	0.5	0.5	0.4	0.4	3.2	4	2.4	3
	Median	1.5	1.5	1.6	1.1	2.5	3.9	2.8	2.3

### Lymph Node Metastases Uptake Patterns

PSMA-11-positive lymph node metastases at any anatomical location (cervical, coeliac, or sacral) were detected in 42.0% (58/138) of the patients. Grouped by their anatomical location, PSMA-11-positive cervical, coeliac, and sacral lymph node metastases were observed at frequencies of 6.5% (9/138 patients), 12.3% (17/138 patients), and 41.3% (57/138 patients; **Figure 1A**). Frequencies of PSMA-11-positive ganglia and lymph node metastases differed at all anatomical locations (P < 0.001; **Figure 1A**).

Qualitatively, among the 83 PSMA-11-positive lymph node, 62.7% (52/83) were identified as FDG-positive and 37.3% (31/83) as FDG-negative (**Figure 1B**). FDG-positive rate in PSMA-11-positive lymph node metastases was higher than in PSMA-11-positive ganglia (62.7% vs 13.6%, P < 0.001).

Quantitatively, the PSMA-11-PET SUVmax ranged from 1.0 to 68.2. No difference was observed in PSMA-11 uptake between the cervical, coeliac, and sacral ganglia (P = 0.316). The FDG-PET SUVmax, which ranged from 0.7 to 23.1, was also similar in the three anatomical locations (P = 0.244; **Table 2**). Representative images for lymph node metastasis are shown in **Figure 2**.

### Comparison of PSMA-11-PET and FDG-PET SUVmax Between Ganglia and Lymph Node Metastases

As shown in **Figure 3**, PSMA-11-PET SUVmax in lymph node metastases was significantly higher than in ganglia (16.4 ± 14.8 vs 2.3 ± 0.7, *P* < 0.001). Similarly, FDG-PET SUVmax in lymph node metastases was significantly higher than in ganglia (3.3 ± 3.2 vs 1.5 ± 0.5, *P* < 0.001).

**Figure 4:**
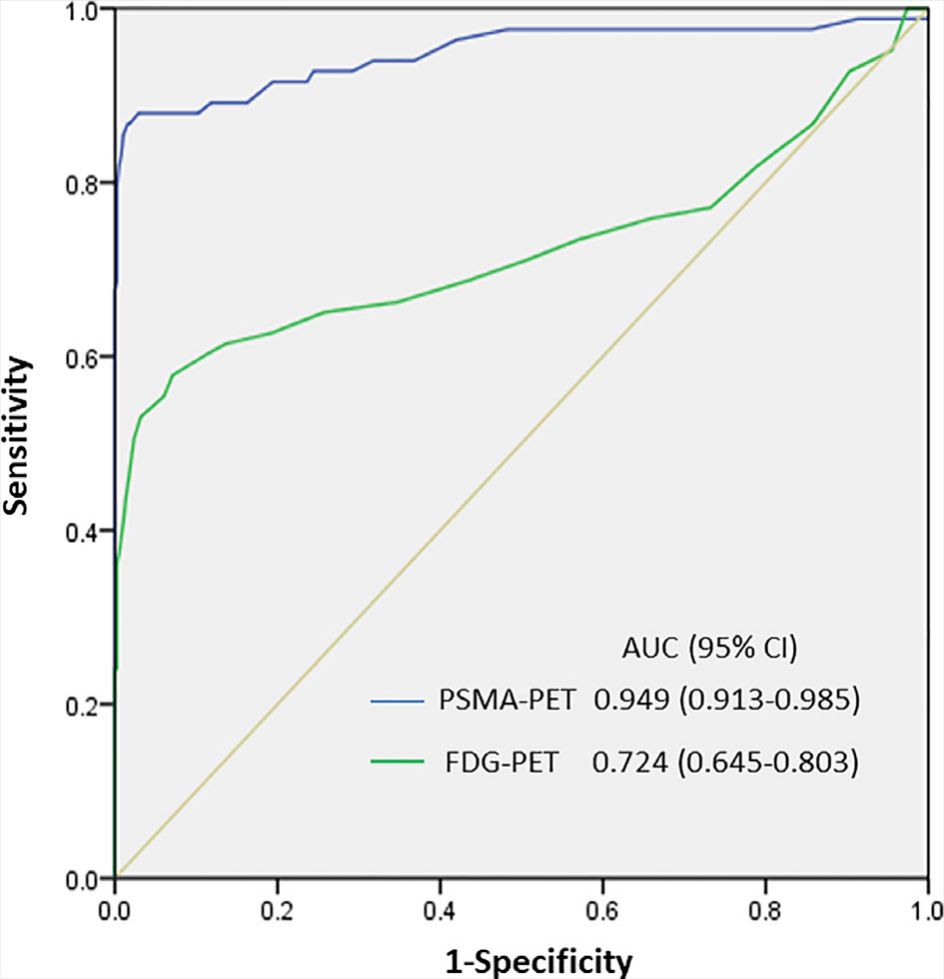
SUVmax of PSMA-11-PET and FDG-PET for distinguishing between lymph node metastasis and ganglia. The area under the curve of PSMA-11-PET was 0.949 (95% confidence interval [CI], 0.913-0.985; *P* < 0.001), and a PSMA-11-PET SUVmax of 4.1 was determined as the optimal threshold for identifying lymph node metastases. With a PSMA-11-PET SUVmax of 4.1, the sensitivity and specificity for identifying lymph node metastases from ganglia were 88.0% (73/83) and 97.1% (370/381), respectively. The area under the curve of FDG-PET was 0.724 (95% CI, 0.645-0.803; *P* < 0.001), and an FDG-PET SUVmax of 2.05 was determined as the optimal threshold for identifying lymph node metastases. With this SUVmax, the sensitivity and specificity for identifying lymph node metastases from ganglia were 60.2% (50/83) and 88.7% (338/381), respectively.

We then determined the optimal PSMA-11-PET or FDG-PET SUVmax thresholds for distinguishing between lymph node metastases and ganglia (**Figure 4**). Receiver-operating characteristic (ROC) curve analysis revealed that when the PSMA-11-PET SUVmax cutoff was 4.1, the sensitivity and specificity for identifying a lymph node metastasis were 88.0% (73/83) and 97.1% (370/381), respectively. The area under curve was 0.949 (95% confidence interval [CI]: 0.913-0.985). Similarly, ROC curve analysis revealed that when the FDG-PET SUVmax cutoff was 2.05, the sensitivity and specificity for identifying a lymph node metastasis were 60.2% (50/83) and 88.7% (338/381), respectively. The area under the curve was 0.724 (95% CI: 0.645-0.803). We further compared the diagnostic performance of PSMA-11-PET and FDG-PET for distinguishing between lymph node metastases and ganglia. PSMA-11-PET SUVmax with an AUC of 0.949 showed a better distinguishing performance than FDG-PET SUVmax with an AUC of 0.724 (*P* < 0.001).

Based on the PSMA-11-PET and FDG-PET SUVmax, we divided the lesions into three groups according to the possibility of them being a lymph node metastasis: a low-potential group (PSMA-11-PET SUVmax of <4.1 and FDG-PET SUVmax of <2.05), moderate-potential group (PSMA-11-PET SUVmax of >4.1 and FDG-PET SUVmax of <2.05 or PSMA-11-PET SUVmax of <4.1 and FDG-PET SUVmax of >2.05), and high-potential group (PSMA-11-PET SUVmax of >4.1 and FDG-PET SUVmax of >2.05). The probabilities of being a lymph node metastasis in the low-, moderate-, and high-potential groups were 0.9% (3/334), 44.6% (37/83), and 91.5% (43/47), respectively (P < 0.001; **Table 3**).

**Table 3:** Rate of being lymph node metastases or ganglia.

Locaiton	Potential	Total (n)	Being lymph node metastases or ganglia		P value
			Lymph node metastases (%)	Ganglia (%)	
**Any location**	Low	334	0.9	99.1	<0.001
	Moderate	83	44.6	55.4	
	High	47	91.5	8.5	
	Total	464	17.9	82.1	
**Cervical and coeliac**	Low	223	0	100	<0.001
	Moderate	53	20.8	79.2	
	High	19	78.9	21.1	
	Total	295	8.8	91.2	
**Sacral**	Low	111	2.7	97.3	<0.001
	Moderate	30	86.7	13.3	
	High	28	100	0	
	Total	169	33.7	66.3	

### Subgroup Analysis According to the Anatomical Location

From the above results, we found that cervical and coeliac ganglia showed higher PSMA-11 and FDG uptake than sacral ganglia (P < 0.001 for all). Lymph node metastases PSMA-11 and FDG uptake were similar in the three anatomical locations. We thus analyzed the lesions according to their anatomical location. With 100% of the lesion being ganglia, we used a PSMA-11-PET SUVmax of <4.1, FDG-PET SUVmax of <2.05 for the cervical and coeliac regions and PSMA-11-PET SUVmax of >4.1, FDG-PET SUVmax of >2.05 for the sacral region.

In the cervical and coeliac regions, the probabilities of being a lymph node metastasis in the low-, moderate-, and high-potential groups were 0% (0/223), 20.8% (11/53), and 78.9% (15/19), respectively (*P* < 0.001; **Table 3** **)**. The probabilities of being a lymph node metastasis in the sacral region in the low-, moderate-, and high-potential groups were 2.7% (3/111), 86.7% (26/30), and 100% (28/28), respectively (*P* < 0.001; **Table 3**).

### The Association Between PSMA-11 or FDG Uptake and the Gleason Score and PSA Level in Ganglia and Lymph Node Metastases

We further investigated whether there was a correlation between PSMA-11 and FDG uptake and the Gleason score or PSA level in ganglia and lymph node metastases.

**Figure 5:**
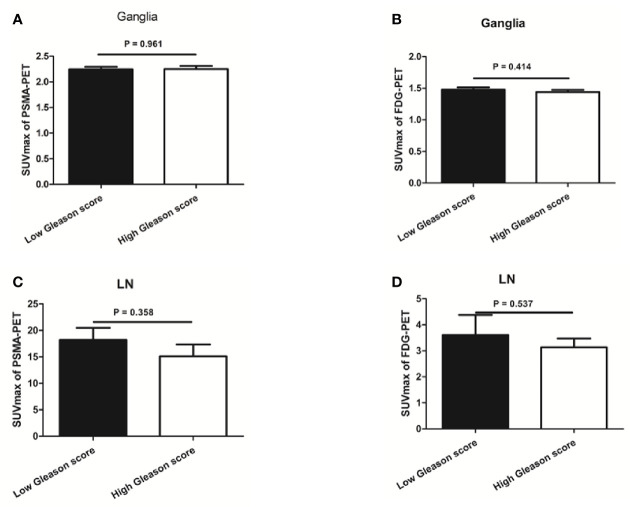
The association between prostate-specific membrane antigen (PSMA-11) or fluorodeoxyglucose (FDG) uptake and Gleason score in ganglia and lymph node (LN) metastases. **(A)** No difference was observed in PSMA-11 uptake between high and low Gleason scores for ganglia (2.3 ± 0.8 vs 2.2 ± 0.7, *P* = 0.961). **(B)** No difference was observed in FDG uptake between high and low Gleason scores for ganglia (1.4 ± 0.4 vs 1.5 ± 0.5, *P* = 0.414). **(C)** No difference was observed in PSMA-11 uptake between high and low Gleason scores for lymph node metastases (15.1 ± 15.0 vs 18.2 ± 13.3, *P* = 0.358). **(D)** No difference was observed in FDG uptake between high and low Gleason scores for lymph node metastases (3.1 ± 2.4 vs 3.6 ± 3.5, *P* = 0.537).

We found no difference in PSMA-11 or FDG uptake between ganglia with high and low Gleason scores (*P* > 0.05 for all, **Figure 5**). Furthermore, no association was found between PSMA-11 or FDG uptake and the PSA level in ganglia of patients evaluated preoperatively (Pearson correlation coefficient between PSMA-11 or FDG uptake and the PSA level: r = 0.115, *P* = 0.401 and r = 0.013, *P* = 0.927, respectively) or following BCR (between PSMA-11 or FDG uptake and the PSA level: r = 0.116, *P* = 0.327 and r = 0.039, *P* = 0.745, respectively).

Similarly, no difference was noted in PSMA-11 or FDG uptake between high and low Gleason scores for lymph node metastases (*P* > 0.05 for both, **Figure 5**). No association was found between PSMA-11 or FDG uptake and the PSA level in lymph node metastases of patients evaluated preoperatively (Pearson correlation coefficient between PSMA-11 or FDG uptake and the PSA level: r = 0.251, *P* = 0.085 and r = 0.137, *P* = 0.564, respectively) or following BCR (between PSMA-11 or FDG uptake and the PSA level: r = 0.042, *P* = 0.831 and r = 0.215, *P* = 0.273, respectively).

## Discussion

Many studies have indicated the unspecific nature of PSMA-11 expression, and PSMA-11-positive ganglia represent a potential diagnostic pitfall for nuclear medicine physicians. In our study, we analyzed the patterns of ^68^Ga-PSMA-11 and ^18^F-FDG tracers uptake by ganglia and lymph node metastases, and whether a dual-tracer PET-CT could be used to tell lymph node metastases and ganglia apart. Our study is the first to describe differences in metabolic patterns in ^68^Ga-PSMA-11 and ^18^F-FDG uptake between ganglia and lymph node metastases, and demonstrate that this difference could be used to tell them apart.

In this study, we identified PSMA-11-positive ganglia in 100% of our patients. These included cervical ganglia in 98.6% of the patients, coeliac ganglia in 96.4%, and sacral ganglia in 81.2%. These results are similar to the PSMA-11-positive rates reported by Rischpler et al. (14). We observed that lymph node metastases had a significantly higher PSMA-11-PET SUVmax than ganglia, which is consistent with other studies (14, 17). PSMA-11 Vinsensia et al. suggested PSMA-11-PET SUVmax of 2.0 as the threshold for PSMA-11-positive lymph node metastases (22). However, our study demonstrated that 60.9% of the ganglia had a PSMA-11-PET SUVmax higher than 2.0. Furthermore, ganglia and lymph node metastases structures can easily be mistaken visually. In a PET-MRI study of coeliac ganglia, Bialek et al. indicated that about half of the patients had at least one ganglion that was confused with PSMA-11-positive lymph node by shape, size, or PSMA-11 uptake (23). Recently, Alberts et al. indicated that delayed ^68^Ga-PSMA-11 PET-CT imaging could be used to differentiate ganglia from lymph node metastases, but the overall diagnostic efficiency of predicting lymph node metastases was not high, with sensitivity and specificity of 73% and 65%, respectively (17). The currently available methods efficiency in differentiating ganglion from lymph node metastasis is not high, so new imaging methods are needed to tell them apart.

We found that among the PSMA-11-positive ganglia and lymph node metastases, 62.7% of the lymph node metastases were FDG-positive, while only 13.6% of the ganglia were FDG-positive. ROC analysis indicated that with an SUVmax cut-off of 2.05, the sensitivity and specificity for predicting a lymph node metastasis were 60.2% and 88.7%, respectively. We also found that the absolute PSMA-11-PET SUVmax in lymph node metastases was significantly higher than in ganglia, which is consistent with previous results (17). PSMA-11We found, based on ROC curve analysis, that an SUVmax cut-off of 4.1 had high sensitivity and specificity, and that PSMA-11-PET SUVmax was better than FDG-PET SUVmax at distinguishing between ganglia and lymph node metastases. The relatively low SUVmax of FDG-PET and PSMA-11-PET for ganglia may be attributed to the low ^18^F-FDG uptake of ganglia and low PSMA-11 expression in ganglia. Because the SUVmax of FDG-PET and PSMA-11-PET for ganglia were lower and narrower that lymph node metastasis, we could distinguish them by the uptake characterization.

We categorized the lesions into three groups based on their potential of being identified as a lymph node metastasis by a combination of PSMA-11-PET and FDG-PET SUVmax. The probability of being a lymph node metastasis was 0.9% in the low-potential group and 91.5% in the high-potential group. Although previous studies indicated that cervical and coeliac ganglia had a higher PSMA-11 uptake than sacral ganglia (14), our study further found that besides PSMA-11 uptake, cervical and coeliac ganglia also had a higher FDG uptake than sacral ganglia. PSMA-11In the sacral region, the probabilities of being a lymph node metastasis in the low-, moderate-, and high-potential groups were 2.7%, 86.7%, and 100%, respectively (*P* < 0.001). PSMA-11In the cervical and coeliac regions, the probabilities of being a lymph node metastasis in the low-, moderate-, and high-potential groups were 0%, 20.8%, and 78.9%, respectively (*P* < 0.001). PSMA-11These results suggest that the pattern of PSMA-11 and FDG uptake by the lesions and their anatomical location should be considered for better differentiation between lymph node metastases and ganglia.

PSA level and Gleason score are independent predictors of PSMA-11 (24) and FDG (18, 19, 25) PET-CT findings. However, PSMA-11no differences were observed in PSMA-11 or FDG uptake between high and low Gleason scores for ganglia. Furthermore, there was also no association between PSMA-11 or FDG uptake and the PSA level for ganglia in patients evaluated preoperatively or following BCR. Similar results were observed with lymph node metastases. Thus, when we differentiate lymph node metastases from ganglia, PSA level and the Gleason score are not risk factors that need to be considered.

Our study has several limitations. The definitions of lymph node metastases and ganglia were made mainly based on their characteristic imaging features, such as typical anatomic location. Pathological evidence was not clinically feasible because of ethical and practical reasons. Although we have established cut-off PSMA-11-PET and FDG-PET SUVmax for telling lymph node metastases from ganglia, this threshold may have been influenced by the PET-CT scanner model, PSMA-11 ligand, scanning procedure, and more. It is essential to establish the optimal SUVmax cut-off in clinical settings according to the actual imaging conditions, and not using PSMA-11-PET SUVmax of 4.1 and FDG-PET SUVmax of 2.05 arbitrarily as the thresholds. Furthermore, the sample size in this study was relatively small, and it was a retrospective study. Therefore, the results could have been influenced by selection bias and should be interpreted carefully. Further prospective studies with more cases are required to confirm our results.

## Conclusions

This is the first study to describe ^68^Ga-PSMA-11 and ^18^F-FDG uptake patterns in ganglia and lymph node metastases. It demonstrates that FDG-PET and PSMA-11-PET SUVmax, especially when data from both tracers is combined, could be used to tell lymph node metastases from ganglia. Differences in ^68^Ga-PSMA-11 and ^18^F-FDG uptake between cervical/coeliac and sacral ganglia suggest that the anatomical location should be considered for better identification.

## Data Availability Statement

The raw data supporting the conclusions of this article will be made available by the authors, without undue reservation.

## Ethics Statement

The studies involving human participants were reviewed and approved by the ethics committee of Renji Hospital. Written informed consent for participation was not required for this study in accordance with the national legislation and the institutional requirements.

## Author Contributions

RC and JL designed and wrote the experiments. RC, LX, YS, and YW collected and analyzed the data. YZ revised the manuscript. All authors contributed to the article and approved the submitted version.

## Funding

The study was supported by National Natural Science Foundation of China (no. 81701724, 81771858, 81530053, 81830052, 81602415, and 81771861).

## Conflict of Interest

The authors declare that the research was conducted in the absence of any commercial or financial relationships that could be construed as a potential conflict of interest.
